# The impact of shyness on social anxiety among college students: Mediating role of regulatory emotional self-efficacy and resilience

**DOI:** 10.1371/journal.pone.0344809

**Published:** 2026-03-11

**Authors:** Yue Wang, Yuan Zhao

**Affiliations:** 1 Faculty of Education and Liberal Arts, INTI International University, Nilai, Malaysia; 2 Police Officer Academy, Shandong University of Political Science and Law, Jinan, China; Golestan University, IRAN, ISLAMIC REPUBLIC OF

## Abstract

**Background:**

Social anxiety is prevalent among college students, and shyness has been consistently linked to elevated social anxiety. However, the psychological mechanisms underlying this association remain insufficiently understood. This study examined whether regulatory emotional self-efficacy and resilience mediate the association between shyness and social anxiety among Chinese college students.

**Methods:**

This study employed the Cheek and Buss Shyness Scale, Regulatory Emotional Self-Efficacy Scale, 10-item Connor-Davidson Resilience Scale, and Interaction Anxiousness Scale to survey 1,012 college students from three universities in Shandong, Anhui, and Jiangsu provinces. Pearson correlation analysis and PROCESS Macro Model 6 regression analysis were used to examine the relationships among shyness, regulatory emotional self-efficacy, resilience, and social anxiety.

**Result:**

Shyness was positively associated with social anxiety. Resilience significantly mediated this association. In addition, shyness was indirectly associated with social anxiety through the sequential pathway of regulatory emotional self-efficacy and resilience. However, regulatory emotional self-efficacy alone did not independently mediate the relationship.

**Conclusion:**

These findings suggest that resilience plays a central role in the association between shyness and social anxiety, and that regulatory emotional self-efficacy may function indirectly through resilience. Interventions targeting resilience and regulatory emotional self-efficacy may help reduce social anxiety among shy college students.

## 1 Introduction

Social anxiety is defined as the worry and fear experienced by individuals in social situations, particularly when they face others’ evaluations, and represents a common form of anxiety [1,2]. Between 7% and 33% of university students worldwide suffer from high levels of social anxiety [3–5]. Research on Chinese college students has indicated that 33.38% of students report having experienced social anxiety symptoms at least once [6]. Social anxiety negatively impacts daily functioning, including close relationships [7], academic performance [8], quality of life [9], and mental health [10]. In educational contexts, particularly in language learning, social anxiety plays a critical role. As language learning is inherently social and communicative, excessive anxiety can reduce learners’ willingness to communicate, limit their participation, and ultimately hinder their language achievement [11].

According to the cognitive-behavioral model of social anxiety [12], the development and maintenance of social anxiety are largely driven by maladaptive cognitive processes, such as negative self-schemas, excessive self-focus, and biased interpretations of social cues. Within this framework, shyness can be viewed as a risk factor that may be associated with increased susceptibility to negative social cognitions, thereby exacerbating social anxiety. Conversely, certain positive psychological and cognitive resources may exert protective effects. Building upon this theory, the present study aims to investigate whether shyness influences college students’ social anxiety through the mediating role of regulatory emotional self-efficacy (RESE) and resilience.

### 1.1 Shyness and social anxiety

Previous studies confirmed that shyness is a key factor contributing to social anxiety [13,14]. Shyness typically refers to the trait of internal nervousness, uneasiness, and excessive self-consciousness that individuals exhibit in social situations [15]. Shy individuals possess the need and motivation to interact with others, but they tend to allocate cognitive resources toward monitoring self-presentation and regulating tension [16]. They spend considerable time during social interactions focusing on their own feelings and behaviors, worrying about leaving a negative impression. This leads to significant fatigue and distress during social engagements, readily triggering social anxiety [17,18]. Research indicates that introverted or shy children and adolescents are more prone to social anxiety, with age differences having no impact on the relationship between shyness and social anxiety [19]. Furthermore, an empirical study by Ran et al. found that shyness is significantly and positively associated with social anxiety among college students aged 18–25, with behavioral inhibition and self-esteem playing crucial roles in mediating this relationship [20]. Based on theoretical and empirical findings, H1 is proposed: Shyness is positively correlated with social anxiety.

### 1.2 Mediating Role of RESE

RESE refers to an individual's level of confidence in regulating their own emotional states [21]. According to self-efficacy theory [22,23], when individuals lack confidence in their ability to regulate negative emotions, they are more likely to perceive themselves as incapable of effectively coping with emotions such as worry, tension, and fear in social situations, thereby increasing social anxiety. Research indicates that RESE is closely linked to social anxiety [24]. That is, individuals possessing higher RESE can effectively alleviate social anxiety [25]. Furthermore, Wan et al. (2024) found that when college students lack confidence in managing negative emotions, they are also unable to employ effective emotional regulation to counteract the negative effects of negative cognition, thereby exacerbating social anxiety [26].

Moreover, shy individuals, due to frequent negative social experiences (such as rejection, embarrassment, or being ignored) or a lack of effective social interaction, tend to hold lower self-evaluations. This may continuously reinforce the cognitive belief that ‘I am unable to effectively regulate my emotions,’ thereby diminishing their RESE [27,28]. Wu et al. (2022) found a significant negative correlation between shyness and RESE [29]. Furthermore, studies by Liu et al. (2018) and Wang et al. (2014) revealed that college students and working adults with higher levels of shyness generally exhibited lower self-efficacy [30,31]. Based on this reasoning, H2 is proposed: The relationship between shyness and social anxiety is mediated by RESE.

### 1.3 Mediating Role of resilience

Resilience is defined as an individual's ability to adapt positively in the face of stress or trauma [32]. It enables individuals to cope with stress, recover from negative experiences, and maintain psychological balance [33]. Resilience, understood as a positive psychological quality, has a beneficial effect on mental health [34,35]. According to the cognitive-behavioral model of social anxiety [12], individuals experience anxiety in social situations primarily due to biased interpretations of threatening cues and negative self-schemas. Resilience helps individuals reduce these negative social cognitions through positive cognitive reappraisal and flexible emotion regulation, thereby lowering social anxiety. Research indicates that among college students, resilience shows a significant negative correlation with social anxiety [36]. Furthermore, Li et al. (2024) found that physical activity can improve social anxiety among college students through the protective effect of resilience [35]. Simultaneously, shyness is closely related to resilience. Shy individuals typically exhibit lower self-esteem [37], and the establishment and maintenance of self-esteem are one of the underlying mechanisms promoting resilience [38]. Moreover, multiple studies have identified a negative correlation between shyness and resilience [39,40], suggesting that shy individuals may possess fewer psychological resources to cope with social stress. Therefore, this leads to H3: The relationship between shyness and social anxiety is mediated by resilience.

### 1.4 Serial Mediation Role of RESE and Resilience

In summary, existing research has predominantly focused on examining the single mediating role of RESE or resilience in the relationship between individual psychological variables and social anxiety, with limited attention given to the sequential mediating effects of RESE and resilience [41]. This limitation restricts our systematic understanding of how shyness influences social anxiety through multiple psychological mechanisms. Previous studies indicate that RESE significantly impacts resilience. Li and Quan (2025) demonstrated that college students can enhance resilience by improving RESE, ultimately reducing adverse mental health outcomes such as depression, anxiety, and stress [42]. Furthermore, Xu and Xu (2025) found that self-efficacy was positively associated with resilience among EFL learners, and this relationship was explained in part by the sequential mediating effects of language anxiety and emotion regulation [43].

According to the cognitive-behavioral model of social anxiety [12], shyness diminishes individuals’ RESE and reduces resilience, thereby increasing social anxiety. Individuals with shyness are susceptible to negative cognitions, leading to lower self-evaluations. This negative experience undermines their belief in their emotional regulation capabilities, thereby reducing RESE [27,28]. Reduced RESE makes it harder for individuals to employ positive strategies to alleviate negative emotions, thereby weakening resilience [44,45]. As a vital psychological resource for coping with adversity and stress, diminished resilience further impairs individuals’ adaptability to social situations [46], making them more prone to developing and sustaining social anxiety [47]. Therefore, H4 is proposed: RESE and resilience sequentially mediate the relationship between shyness and social anxiety.

**Fig 1:**
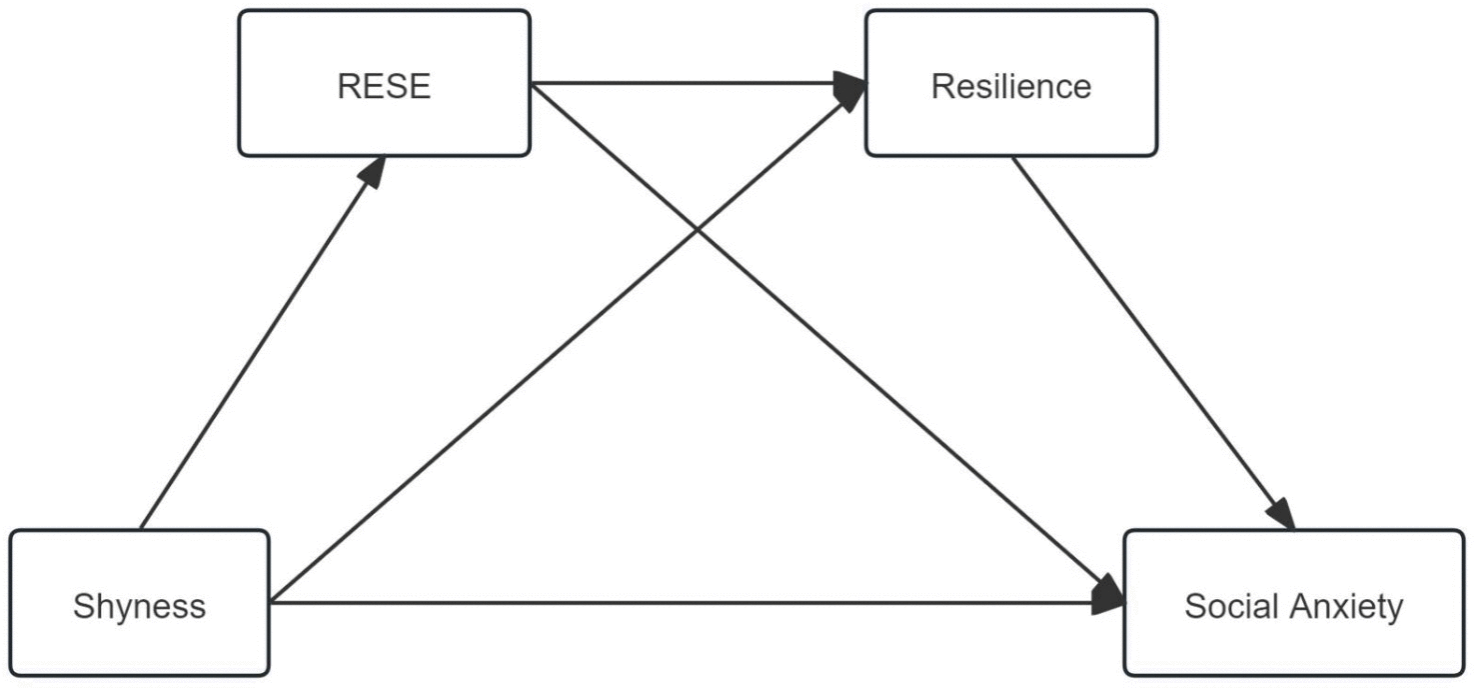
Conceptual framework of the serial mediation model.

Shyness, RESE, resilience, and social anxiety are interrelated constructs. However, the mechanisms through which these variables are associated within a unified framework remain insufficiently understood in the college student population. This study proposes a conceptual framework (Fig 1) to examine the association between shyness and social anxiety from the perspective of college students, and to explore the potential mediating role of RESE and resilience. This framework aims to provide theoretical support and guidance for research and interventions related to the psychological and behavioral health of college students. Collectively, this study proposes four research hypotheses:

H1: Shyness is positively correlated with social anxiety.
H2: The relationship between shyness and social anxiety is mediated by RESE.
H3: The relationship between shyness and social anxiety is mediated by resilience.
H4: RESE and resilience sequentially mediate the relationship between shyness and social anxiety.

## 2 Methods

### 2.1 Participants

From May 6 to July 5, 2024, the study employed convenience sampling to recruit participants from three universities in Shandong, Anhui, and Jiangsu provinces. First, we recruited five counselors from each institution to serve as survey administrators. These administrators then distributed online questionnaires to students via WeChat or QQ groups, inviting voluntary participation. The minimum sample size was 129 based on a medium effect size of 0.15, α of 0.05 and a power of 0.95 (using G*power 3.1 version) [48]. A total of 1,012 valid questionnaires were collected, with the sample covering 12 provinces, municipalities, and autonomous regions including Shandong, Anhui, Jiangsu, and Sichuan. After data processing, 47 questionnaires were excluded due to missing key research variables, logical errors, or insufficient response time. Ultimately, 965 valid samples were identified, achieving a validity rate of 95.36%. The sample size adequately met research requirements, comprising 511 male and 454 female students. Prior to completing demographic measurements and questionnaires, all participants signed informed consent forms indicating voluntary participation. The entire data collection process strictly adhered to principles of voluntariness and confidentiality. All participants were informed they could refuse to answer any uncomfortable questions or withdraw from the study at any time. Each completed questionnaire was compensated with 1 RMB. This study was reviewed and approved by the Ethics Committee of the Police Officer College, Shandong University of Political Science and Law.

### 2.2 Materials and measures

#### 2.2.1 Revised cheek and buss shyness scale (RCBS).

The RCBS, originally developed by Cheek [49], was subsequently revised by Xiang [50] and evaluated for reliability and validity among a sample of Chinese university students. When revising the questionnaire, it was translated according to Chinese culture. The scale is scored using a 5-point Likert scale, with 1 indicating “not at all true” and 5 indicating “extremely true.” Once the items have been reversed, the scores for each item are totaled. A higher total score indicates a stronger sense of shyness. In this study, Cronbach's α was found to be 0.93.

#### 2.2.2 Regulatory emotional self-efficacy scale.

This study employed the Chinese version of the RESE, developed by Caprara and revised by Zhang et al. [51]. The scale comprises 12 items across three dimensions: efficacy in expressing positive emotions (Items 1–4), efficacy in regulating distress/painful emotions (Items 5–8), and efficacy in regulating anger/irritability (Items 9–12). The scale employs a 5-point Likert scale ranging from 1 (strongly disagree) to 5 (strongly agree). Previous research has demonstrated its strong construct validity and reliability among Chinese university students. Higher scores indicate greater self-efficacy in emotion regulation, reflecting greater confidence and perceived effectiveness in managing and regulating diverse emotions. In this study, the Cronbach's α coefficient for the scale was 0.95.

#### 2.2.3 10-item Connor-Davidson Resilience Scale (CD-RISC-10).

This scale was simplified from the 25-item CD-RISC by Campbell‐Sills and Stein (2007) and is currently widely used in related research. The reliability and validity of the scale have been well demonstrated in Chinese populations [33,52]. The scale comprises 10 items. Scoring is conducted using a Likert 5-point scale, where 1 indicates “never like this” and 5 represents “always like this.” A higher number indicates greater conformity to the statement in question. A higher total score indicates an elevated level of resilience. In this study, Cronbach's α was 0.93.

#### 2.2.4 Interaction Anxiousness Scale (IAS).

The IAS, initially developed by Leary (1983) and subsequently revised by Peng et al. (2004), has been demonstrated to be an efficacious instrument for investigating social anxiety among university students in China [53,54]. The total scale consists of 15 items. A five-point Likert scale is employed, with 1 indicating “not at all true” and 5 indicating “extremely true.” A higher total score reflects an elevated level of social interaction for the individual. In this study, the Cronbach’s α was 0.93.

### 2.3 Statistical analyses

This study employed SPSS 26.0 software to conduct a series of statistical analyses. Prior to formal analysis, Harman's single-factor test and single-factor confirmatory factor analysis were first applied to examine common method bias among the study variables. Subsequently, confirmatory factor analysis was used to further evaluate the structural validity, convergent validity, and reliability of the measurement model. Descriptive statistics and Pearson correlation analyses were then conducted to preliminarily explore relationships among variables. To examine mediating effects, Hayes’ PROCESS macro (Model 6) analyzed the sequential mediation model between shyness and social anxiety, focusing on the mediating roles of regulatory emotional self-efficacy (RESE) and resilience. Finally, confidence intervals (CI) were generated using 5000 bootstrap samples to test the significance of mediating effects. Effects were considered significant when the CI did not include zero. P-values were two-tailed and considered statistically significant at <0.05.

## 3 Results

### 3.1 Common method deviation test

Since all data in this study were collected via self-reported questionnaires, we employed Harman's single-factor test and single-factor confirmatory factor analysis to examine common method bias. Results revealed 19 factors with characteristic roots greater than 1, with the first factor explaining 29.08% of total variance, below the 40% critical threshold. Further single-factor confirmatory factor analysis revealed poor model fit indices (χ²/df = 15.52, CFI = 0.43, TLI = 0.41, RMSEA = 0.12), indicating the single-factor model was not well-suited to the data structure. Collectively, these findings suggested no severe common method bias exists in the study data.

### 3.2 Confirmatory factor analysis

To validate the construct validity of the measurement tools, this study conducted confirmatory factor analysis (CFA) on four latent variables. Results indicated that the measurement model fit the data well (χ² = 3026, χ²/df = 2.59, CFI = 0.94, TLI = 0.94, RMSEA = 0.041). All measurement items exhibited significant standardized factor loadings ranging from 0.72 to 0.97, exceeding the recommended threshold of 0.50. Further calculations revealed that the composite reliability (CR) of each latent variable exceeded 0.90, and the average variance extracted (AVE) exceeded 0.60, as shown in Table 1. Therefore, the measurement model in this study was considered to possess good convergent validity and reliability.

**Table 1:** AVE and CR for study variables.

Variables	AVE	CR
**1 Shyness**	0.71	0.97
**2 RESE**	0.70	0.96
**3 Resilience**	0.76	0.97
**4 Social Anxiety**	0.68	0.97

RESE, Regulatory Emotional Self-efficacy.

### 3.3 Descriptive analysis and correlation analysis

As shown in Table 2, the study variables exhibited significant correlations, providing preliminary support for our hypotheses. Preliminary analyses indicated that gender (0 = female, 1 = male) and age (*M ± SD* = 20.44 ± 1.70) were not significantly correlated with the main study variables (all *p*s > 0.18).

**Table 2:** Means, standard deviations, and correlations for study variables.

Variable	*M* ± *SD*	1	2	3	4
**1 Shyness**	45.86 ± 11.33	1			
**2 RESE**	38.32 ± 10.29	−0.17^***^	1		
**3 Resilience**	14.52 ± 9.02	−0.43^***^	0.46^***^	1	
**4 Social Anxiety**	53.41 ± 12.76	0.39^***^	−0.17^***^	−0.42^***^	1

*N* = 965. *p < 0.05, **p < 0.01, ***p < 0.001, same as below.

### 3.4 Test of mediation effect

**Fig 2:**
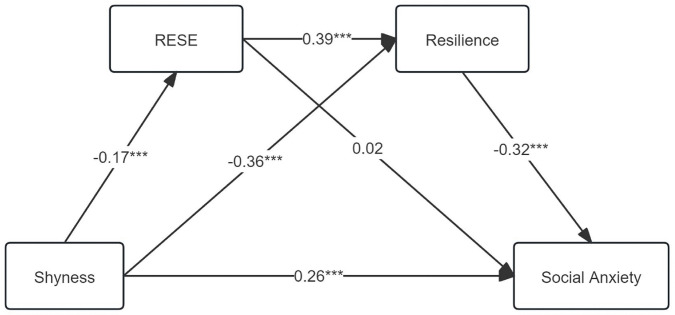
Mediating effect model.

After data standardization using SPSS 26.0, regression analysis was conducted on the mediation hypotheses using Model 6 of the Process plugin developed by Hayes. Results were presented in Table 3 and Fig 2. Shyness is significantly negatively correlated to RESE (*β* = −0.17, *p* < 0.001) and resilience (*β* = −0.36, *p* < 0.001). However, shyness is significantly positively correlated to social anxiety (*β* = 0.26, *p <* 0.001). RESE is significantly positively correlated to resilience (*β* = 0.39, *p* < 0.01). However, RESE was unrelated to social anxiety (*β* = 0.02, *p* > 0.05). Resilience is significantly negatively correlated to social anxiety (*β* = −0.32, *p* < 0.001).

**Table 3:** Serial mediation model for regression-based analysis.

Outcome Variable	Predictive Variable	*R*	*R* ^*2*^	*F*	*β*	*t*	LLCI	ULCI
**RESE**	**Shyness**	0.17	0.03	29.81^***^	−0.17^***^	−5.46	−0.21	−0.10
**Resilience**	**Shyness**	0.58	0.34	244.20^***^	−0.36^***^	−13.62	−0.33	−0.25
	**RESE**				0.39^***^	14.78	0.30	0.39
**SA**	**Shyness**	0.48	0.23	97.12^***^	0.26^***^	8.31	0.22	0.36
	**RESE**				0.02	0.75	−0.05	0.11
	**Resilience**				−0.32^***^	−9.19	−0.55	−0.35

RESE, Regulatory Emotional Self-efficacy; SA, Social Anxiety.

*p  <  0.05, **p  <  0.01, ***p  <  0.001 (two-tailed).

The mediating effects of RESE and resilience on the relationship between shyness and social anxiety were examined using percentile bootstrap tests with bias correction, and 95% confidence intervals were calculated. Results were presented in Fig 2 and Table 4. Resilience significantly mediated the association between shyness and social anxiety. In addition, the serial mediation of RESE and resilience was significant. However, the indirect effect of RESE alone was not significant.

**Table 4:** Decomposition of direct effect, mediation effect and total effect.

	*B*	*SE*	95% CI		Effect (%)
			BootLLCI	BootULCI	
**Total effect**	0.44	0.03	0.38	0.51	
**Direct effect**	0.29	0.04	0.22	0.36	65.91%
**Total indirect effect**	0.15	0.02	0.11	0.19	34.09%
**Shyness → RESE → SA**	−0.01	0.01	−0.02	0.01	−0.01%
**Shyness → Resilience → SA**	0.13	0.02	0.10	0.17	29.55%
**Shyness → RESE → Resilience → SA**	0.02	0.01	0.01	0.04	4.55%

RESE, Regulatory Emotional Self-efficacy; SA, Social Anxiety.

Additionally, the direct effect of shyness on social anxiety was 0.29, accounting for 65.91% of the total effect. Moreover, the indirect pathway via resilience accounted for 29.55% of the total effect, whereas the sequential pathway through RESE and resilience explained 4.55% of the total effect. These findings supported H1, H3, and H4. However, the indirect pathway from shyness to social anxiety through RESE alone was not significant, as the 95% CI included zero, indicating that RESE did not independently mediate this association. Therefore, H2 was not supported.

## 4 Discussion

This study systematically examined the associations among shyness, RESE, resilience, and social anxiety based on a sample of 965 Chinese university students. Findings indicated that shyness was positively associated with social anxiety. In addition, shyness was linked to social anxiety through the mediating effect of resilience and the sequential mediating effect of RESE and resilience, whereas RESE alone did not independently mediate this association. Comparative analyses further showed that resilience accounted for the largest proportion of the indirect effect. These findings contribute to the existing literature by clarifying how personality characteristics and psychological resources are jointly associated with social anxiety among college students. By integrating shyness, RESE, and resilience within a unified analytical framework, the present study offers a more nuanced understanding of the mechanisms underlying social anxiety and provides meaningful implications for the development and refinement of intervention strategies.

The present study identified a significant positive association between shyness and social anxiety among college students, supporting H1. This finding is consistent with prior research indicating a close link between shyness and elevated levels of social anxiety, and suggests that shyness may function as a vulnerability-related trait associated with increased social anxiety [13,18,20]. Accordingly, the present study provides further empirical evidence for the association between shyness and social anxiety within a college student population.

The present findings suggest that resilience serves as a significant mediator in the association between shyness and social anxiety, supporting H3. The findings indicate a significant negative correlation between shyness and resilience among college students, consistent with previous research [39,40]. Individuals with higher shyness exhibit avoidance behaviors in social settings, experience negative emotions such as tension and unease, and tend to avoid novel or unfamiliar social environments [18]. Furthermore, shyness is associated with poorer social adaptation abilities [55], social skills [56], and emotional regulation [57], which may be reflected in lower levels of resilience. Additionally, this study indicates a significant negative correlation between resilience and social anxiety, consistent with prior research. Li and Zheng (2025) found that higher resilience correlates with lower social anxiety [58]. Specifically, individuals with greater resilience are more adept at regulating their emotions and employing rational coping strategies to manage their social anxiety. Furthermore, research indicates that resilience mediates the relationship between social exclusion and social anxiety, buffering their connection [59]. Thus, shy college students can alleviate social anxiety by enhancing their resilience.

However, the findings of this study revealed that RESE did not significantly mediate the relationship between shyness and social anxiety, thus failing to support H2. One possible explanation is that the influence of regulatory emotional self-efficacy on social anxiety may be time-dependent. RESE reflects an individual’s perceived capability to manage negative emotions [21], yet its protective effect may accumulate gradually through repeated emotional regulation experiences rather than exerting an immediate influence [60]. Given the cross-sectional design of the present study, such dynamic processes may not have been adequately captured.

Measurement considerations may also contribute to this finding. RESE assesses perceived emotional regulation competence rather than actual regulatory performance. It is possible that individuals with high shyness still perceive themselves as capable of emotion regulation, but such perceived competence may not necessarily translate into effective regulation in socially threatening contexts [61,62]. Thus, the discrepancy between perceived and enacted regulation could attenuate its direct association with social anxiety.

In addition, contextual factors may condition the effectiveness of RESE. In highly evaluative or socially demanding environments, resilience may play a more proximal and robust role in buffering social anxiety, whereas RESE may exert its influence indirectly by enhancing resilience over time. This interpretation is consistent with prior research suggesting that RESE may reduce psychological distress primarily through indirect or sequential pathways rather than through direct effects [63].

Finally, this study also found that RESE and resilience sequentially mediated the relationship between shyness and social anxiety among college students, validating H4. This result indicates that individuals with shyness often exhibit deficits in emotional regulation confidence [64], making them prone to perceive difficulties in effectively managing and expressing emotions, which consequently leads to lower levels of resilience [42]. As a psychological resource for coping with stress and challenges, insufficient resilience weakens an individual's adaptive capacity and coping strategies in social situations, which may be associated with greater tendencies toward social avoidance and higher levels of social anxiety [35]. This finding aligns with the cognitive-behavioral model of social anxiety. Consequently, this study offers a novel perspective for exploring the underlying mechanisms through which shyness influences social anxiety among college students. Specifically, RESE and resilience can be regarded as key psychological resource variables within the cognitive model. Their absence or insufficiency may be linked to heightened negative self-processing and threat perception among shy individuals, thereby maintaining or exacerbating social anxiety.

Building on these findings, the present study offers several practical implications for the prevention and intervention of social anxiety among college students. First, the robust mediating role of resilience suggests that interventions aimed at strengthening resilience may be particularly effective in reducing social anxiety. Programs that emphasize adaptive coping strategies, stress management, cognitive reappraisal, and problem-solving skills can enhance students’ capacity to navigate socially challenging situations. By fostering psychological flexibility and effective coping, such interventions may buffer the impact of shyness on social anxiety. Second, although RESE did not independently mediate this relationship, its role within the sequential pathway indicates that enhancing RESE may indirectly contribute to greater resilience. Interventions that incorporate emotion regulation training, such as mindfulness-based practices, cognitive restructuring, and emotional expression skills training, may bolster students’ confidence in managing their emotional experiences. Over time, strengthened RESE may help build resilience, thereby lowering vulnerability to social anxiety. Finally, intervention efforts should also consider the broader university context. Cultivating supportive peer networks, reducing excessive evaluative pressure, and promoting an inclusive campus climate can provide external protective resources that complement individual-level psychological interventions.

## 5 Limitations and future study

This study has several limitations. First, the cross-sectional design precludes causal inferences among the variables. Second, the use of convenience sampling and self-reported measures may introduce social desirability bias and common method bias. Third, the sample was limited to the eastern provinces of China, which may restrict the generalizability of the findings to other regions. Finally, the potential role of cultural factors was not examined in depth. Future research could employ longitudinal or experimental designs to strengthen causal inference, adopt probability sampling and multi-source data to enhance representativeness and reduce self-report bias, expand the geographic scope of the sample, and further investigate the potential influence of cultural factors on the relationships among the variables.

## 6 Conclusion

In summary, the following conclusions were reached: (1) Shyness and social anxiety among college students are positively correlated; (2) RESE does not serve as an independent mediator between shyness and social anxiety; (3) Resilience mediates the relationship between shyness and social anxiety; (4) Shyness indirectly influences social anxiety through the serial mediating effects of RESE and resilience. These findings fill a gap in research on the underlying psychological mechanisms through which shyness exacerbates social anxiety among college students, while also providing empirical support for future mental health education and intervention strategies in higher education settings. Specifically, enhancing students’ regulatory emotional self-efficacy and resilience may serve as effective pathways to reduce social anxiety levels in shy individuals, thereby offering theoretical foundations for practical applications such as psychological counseling, group counseling, and curriculum design.
